# Evaluating Professional Networks in a Gerontological Pre-Dissertation Initiative Using Social Network Analysis

**DOI:** 10.1093/geroni/igaa057.1797

**Published:** 2020-12-16

**Authors:** Erin Murphy, Rebecca Mauldin, Jennifer Greenfield, Nancy Kusmaul, Noelle Fields, Stephanie Wladkowski, Allison Gibson

**Affiliations:** 1 UT Arlington, School of Social Work, Arlington, Texas, United States; 2 University of Texas at Arlington, Arlington, Texas, United States; 3 University of Denver, Denver, Colorado, United States; 4 UMBC, Baltimore, Maryland, United States; 5 The University of Texas at Arlington, Arlington, Texas, United States; 6 Eastern MIchigan University, Ypsilanti, Michigan, United States; 7 University of Kentucky, College of Social Work, Lexington, Kentucky, United States

## Abstract

Professional networks are critical for PhD students and early career faculty, yet there is scant research on the development of their professional networks. Social network analysis is a useful approach to describe the development of professional networks. This methodological paper explains its use and benefits, using a social network analysis of alumni from the first three cohorts of the Association of Gerontological Education in Social Work (AGESW)’s Pre-Dissertation Fellowship Program (PDFP) as an example. We present results, challenges, and recommendations. Alumni (n = 12) reported meeting an average of 20 scholars (SD = 13.2) through AGESW. These professional relationships led to collaborations on conference presentations and manuscripts as well as opportunities to leverage the relationships for future professional needs. Suggested applications of social network analysis for program evaluation, such as co-author and citation networks, are also presented with a focus on training programs designed to support robust professional network development.

